# Synergistic effect of N-decorated and Mn^2+^ doped ZnO nanofibers with enhanced photocatalytic activity

**DOI:** 10.1038/srep32711

**Published:** 2016-09-07

**Authors:** Yuting Wang, Jing Cheng, Suye Yu, Enric Juan Alcocer, Muhammad Shahid, Ziyuan Wang, Wei Pan

**Affiliations:** 1 State Key Laboratory of New Ceramics and Fine Processing, School of Materials Science and Engineering, Tsinghua University, Beijing 100084, People’s Republic of China; 2 Department of Physics, University of Science and Technology Beijing, Beijing 100083, China; 3 Department of materials, Imperial College London, Exhibition Road, London SW7 2AZ, United Kingdom

## Abstract

Here we report a high efficiency photocatalyst, i.e., Mn^2+^-doped and N-decorated ZnO nanofibers (NFs) enriched with vacancy defects, fabricated via electrospinning and a subsequent controlled annealing process. This nanocatalyst exhibits excellent visible-light photocatalytic activity and an apparent quantum efficiency up to 12.77%, which is 50 times higher than that of pure ZnO. It also demonstrates good stability and durability in repeated photocatalytic degradation experiments. A comprehensive structural analysis shows that high density of oxygen vacancies and nitrogen are introduced into the nanofibers surface. Hence, the significant enhanced visible photocatalytic properties for Mn-ZnO NFs are due to the synergetic effects of both Mn^2+^ doping and N decorated. Further investigations exhibit that the Mn^2+^-doping facilitates the formation of N-decorated and surface defects when annealing in N_2_ atmosphere. N doping induce the huge band gap decrease and thus significantly enhance the absorption of ZnO nanofibers in the range of visible-light. Overall, this paper provides a new approach to fabricate visible-light nanocatalysts using both doping and annealing under anoxic ambient.

Recently, increasing environmental pollution provides impetus for sustained research in efficient catalysts which can use sunlight to decompose or oxidize the organic pollutants^1^. As a potential photocatalytic material, ZnO has received great attention because of its wide band gap (3.37 eV) and large exaction binding energy (60 meV)^2^,^3^, but it can absorbs light only in the ultraviolet (UV) range, greatly limiting its overall solar-to-fuel efficiency. Much research has been carried out to enhance the visible-light photocatalytic activity of ZnO by doping or incorporating. Moreover, researchers have attempted to increase the surface to volume ratio for improving photocatalytic activity^4^. They found that nanofibers have an even higher catalytic activity than nanoparticles and bulk forms due to their crystallinity and high surface to volume ratio^5^. Further research shows the density of surface defects would be more deterministic rather than the specific surface area^6^, in which intrinsic defects introduce more mid-gap states and largely enhance the transition probability of the valence electrons to its conduction band. Thus, in photocatalysis, oxygen vacancies and surface defects are also involved in optimizing electronic band structure. Besides, the charged oxygen vacancies are beneficial for electron transfer and electron-hole pair separation, contributing to a significant increase in photocatalytic efficiency^7^,^8^. Surface disorder and point defects can be created intentionally within the band gap through reduction/crystallization process. Naldoni *et al.*^9^ demonstrate that black TiO_2_ nanoparticles with crystalline core/disordered shell morphology exhibit a narrowed bandgap due to the synergistic presence of oxygen vacancies and surface disorder. As an impurity band, the oxygen vacancy states, located between the valence and conduction bands, could narrow the band gap of a semiconductor, facilitating the absorption and excitation of photons under visible light^10^. Many researches focused on the excellent visible-light catalytic performance of nano-TiO_2_ decorating by oxygen vacancy^11^,^12^,^13^,^14^. However, there are only few studies of ZnO showing good photocatalytic efficiency in visible-light range^15^,^16^.

Another important feature in catalytic performance is that there is no driving force to trap electrons on doping centers and hence they will have better photoconductivity. A high spin d^4^ ion like Mn^3+^, the tremendous intra-atomic exchange energy gained upon converting to a d^5^ configuration provides an enormous driving force to trap electrons that are diffusing nearby. By contrast, d^0^, d^10^, and high spin d^5^ ions gain no exchange energy by trapping an electron (e.g., Fe(III), Mn(II), and Cu(I)), which makes them the ideal candidates for active dopants. Among the metal ions, Mn^2+^ ions with half-filled electronic configuration show excellent catalytic activity. They could trap the charge carriers shallowly and efficiently to facilitate the detrapping process of charge carriers to the surface of the catalyst which accelerate the interfacial charge transfer processes^17^. Moreover, previous studies of N-doped TiO_2_ or ZnO have shown a significant red shift in light absorption wavelength and notable improvement in photocatalytic activity^14^,^18^,^19^. Here we report a significant enhancement in visible-light photocatalytic activity of ZnO by creating intentional defects in its crystal lattice via Mn^2+^-doping and N_2_ atmosphere annealing. Mn^2+^ was chosen because it will not act as a recombination center but will create more intermediate states.

To date, various methods have been employed to obtain high-performance photocatalysts, such as hydrothermal^20^, sol-gel^21^, microwave irradiation^16^, chemical precipitation^22^, electrospinning^23^, *etc*. Among these approaches, electrospinning appears to be the most facile and practical technique for manufacturing nanofibers with small crystal size^23^,^24^. This technique performance is not only feasible and simple but also it is an ideal way to prepare nano-materials with specific composition, large specific surface area and recoverable character. It is also reported that an increasing number of nanocatalysts are fabricated by this approach^25^,^26^,^27^. Some notable examples include ZnO, TiO_2_, SnO_2_, ZnS and BiVO_4_^1^,^28^,^29^,^30^,^31^, *etc*.

Hence, in this research, a simple and efficient electrospinning method combined with the controlled heat treatment were applied for the synthesis of Mn^2+^-doped and N-decorated ZnO nanofibers. The morphologies, crystalline structures, optical properties, and photocatalytic performances for all samples were explored to clarify the correlation between nanostructures and photocatalytic properties. Furthermore, underlying mechanisms responsible for this novel photocatalytic material have been proposed. The ensemble of results showed that enhanced photocatalytic performance and excellent efficiency are owing to both the Mn^2+^ and N doping, which induce large amounts of surface defects. It is envisioned that our work may provide a new approach for preparing nano-sized ion- and N-doped ZnO photocatalysts with high density of oxygen vacancies.

## Results

### Characterization of Mn-ZnO nanofibers

**Figure 1:**
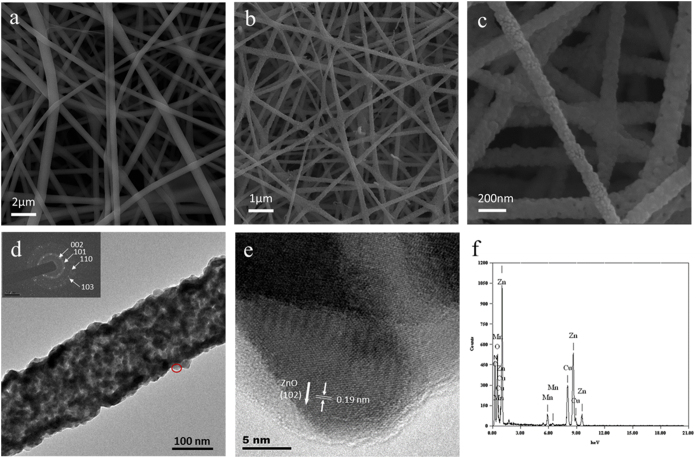
SEM images of (**a**) as-spun 15% Mn^2+^-doped ZnO nanofibers, (**b**) after annealing at 550 °C for 1 h in N_2_, (**c**) enlarged view of the annealed nanofibers. (**d**) TEM image of 15% Mn^2+^-doped ZnO nanofibers. The inset shows the SAED pattern. (**e**) HRTEM image taken from the nanofiber indicated by the ellipse in (**d**). (**f**) EDS spectra from the single nanofiber.

Figure 1a shows the SEM images of the as-spun 0.15 Mn-ZnO nanofibers with smooth surface and a range of diameters about 300 nm. By using electrospinning, all the fibers collected on the aluminum substrate show uniform and high density. After the as-spun fibers calcined for 1 h under inert atmosphere, the continuous structure of the fibers were maintained, and the surface became rough. The diameter after annealing reduces to 100 ± 20 nm (see Fig. 1b,c).

**Figure 2:**
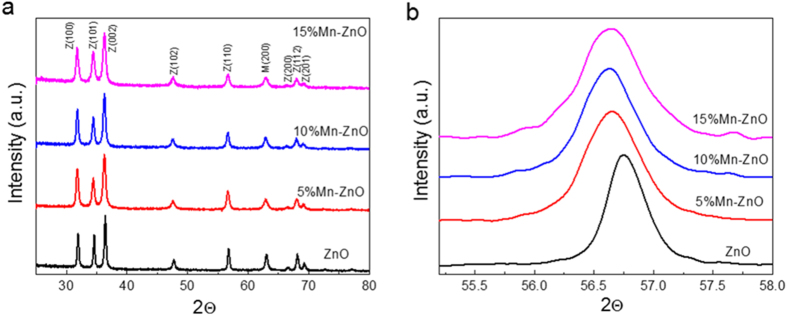
(**a**) XRD patterns of 5%, 10% and 15% Mn^2+^-doped ZnO nanofibers. (**b**) The step-scanning XRD of all the nanofibers.

To further investigate the internal structure, the nanofibers were dispersed in ethanol evenly and viewed under TEM. It is obvious that the nanofiber shows a fibrillar structure after annealing. A selected-area electron diffraction (SAED) pattern reveals that the nanofibers are comprised of polycrystalline and fine grains of ZnO with no preferential orientation. The intensity of the wurtzite rings is weak, however, the presence of (002) (101) (100) and (103) lattice spacings can be resolved. As shown in Fig. 2e, recorded from the area marked by the red circle in Fig. 2d, the high resolution TEM (HRTEM) image indicates that the lattice constants between two adjacent parallel atomic lattice-fringes are 0.19 nm, which can be attributed to the ZnO (102) plane. Both the SAED and HRTEM results demonstrate the annealed 0.15 Mn-ZnO nanofibers are composed of only hexagonal ZnO and no other phases, such as MnO or MnO_2_. Furthermore, Energy-dispersive X-ray spectroscopy (EDS) analysis confirms the concentration of Mn is found to be 14.85 at.% (Fig. 2f).

The phase composition and structure of the annealed samples were examined by X-ray diffraction (XRD). Figure 2 shows XRD patterns of a series of Mn-ZnO nanofibers with different concentrations of Mn^2+^ ions annealed at 550 °C. The main diffraction peaks are indexed to hexagonal wurtzite phases, and there is no other manganese oxide phase, which is in accord with the TEM result. All the diffraction peaks fit well with the hexagonal phases, as labeled in Fig. 2a, manifesting Mn atoms were successfully doped into the lattice. Since Mn easily donates its electron to O, the preferable site of substitution of Mn in ZnO lattice is Zn site. However, XRD spectra is unable to detect a crystal structure if the content of it is below 3%. Thus, an excess Mn^2+^ doping have done to identify the saturated concentration of Mn^2+^, as shown in Figure S1. The crystalline structures corresponding to MnO and ZnO phases are confirmed, and no other peak relates to manganese compound with high valence is detected. Moreover, the concentration of these two phases were measured by the quantitative analysis using jade 8. The concentration of MnO phases in 0.5 Mn-ZnO sample is about 30 at.%, which indicates nearly 30 at.% Mn^2+^ could be loaded in ZnO lattice. Thus, all Mn^2+^ atoms were successfully doped in the lattice even after 15 at.% loading in this study. Moreover, it is noted that the diffraction peaks for Mn-ZnO samples gradually shift to lower angles with the increase of Mn concentration. Clearly, it could also be seen from the step scanning XRD patterns that the single diffraction peak in the range of 55.2–58° shifts to lower angles (Fig. 2b). With increasing Mn content, a clear increase of the lattice constants could be observed from the results of lattice calculation (Supplementary Table S1). Since the Mn^2+^ ions occupy the positions of the Zn^2+^ ions, the lattice parameters and cell volume accompany with an increase, leading to a shift to lower diffraction angles of XRD peaks. As the ionic radius of Mn^2+^ (80 pm) is larger than that of lattice Zn^2+^ (74 pm), the hypothetic Mn^4+^ (67 pm) doping could be excluded^32^,^33^.

### Photocatalytic Activity and Stability

**Figure 3:**
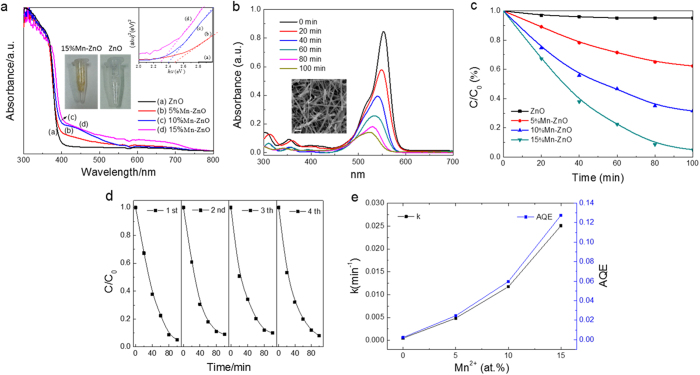
(**a**) UV−vis diffuse reflectance spectra (DRS) of undoped and Mn^2+^-doped ZnO fibers. The inset shows the corresponding plots of (αhν)2 versus photon energy (hν). (**b**) UV-vis absorption spectra of RhB at different time in the presence of 15% Mn^2+^-doped ZnO nanofibers under visible light. The inset illustrates the SEM image of the specimen reclaimed after photocatalytic measurement. (**c**) Photodegradation of RhB by ZnO nanofibers doped with different Mn^2+^ concentration. (**d**) Cycling tests of photodegradation of the specimen. (**e**) Degradation rate constants and apparent quantum efficiencies of ZnO nanofibers with different doping concentrations.

The photocatalytic activities of the nanofibers with different Mn concentration were evaluated by the degradation of RhB dyes under visible light irradiation (400 < λ < 750). Temporal changes in the concentrations of RhB were monitored by examining the variations in the maximal absorption peak at 554 nm in the UV−vis spectra. The total optical power impinging on the solution is 100 mW mL^−1^, and the effective surface area of the nanofibers measured by BET test is 18.86 ± 2.23 m^2^/g. Figure 3b shows the photocatalytic removal of the RhB using 0.15 Mn-ZnO nanofibers under visible light for 100 min. The inset of Fig. 3b exhibits SEM images of the nanofibers after photocatalytic measurement, which displays the catalysts still keep their three-dimensional structure but some are broken up into pieces during ultrasonic suspension. Comparative experiments were also carried out to investigate the photocatalytic activity of the nanofibers with different Mn composites under identical conditions. In Fig. 3c, when Mn^2+^ was doped in ZnO nanofibers, a remarkable enhancement in photocatalytic activity is observed clearly. The optimal loading is around 15 at.%, since RhB is completely degraded after irradiating 100 min. The RhB adsorbed on the sample surface has also been proved to be removed totally (see FTIR results, Supporting Information Figure S2). There are no observable changes of the peaks in the 0.15 Mn-ZnO nanofibers before and after photocatalytic cycles. Hence, not only the photocatalytic activity of ZnO nanofibers is highly enhanced by Mn^2+^ doping, they also show a stable photocatalytic activity under visible light. Figure 3d displays no decay of this sample for photocatalytic activity within several cycles. As known, the photodegradation of RhB can be considered as a pseudo-first-order process^34^,^35^, and its kinetics can be described by the formula as follows:


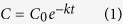


where *C* is the concentration of RhB that at the reaction time t, *C*_0_ is the initial concentrations of RhB, and *k* is the degradation rate constant. The 0.15 Mn-ZnO nanofibers exhibit the highest catalytic activity with constant rate (*k*) of 2.5 × 10^−2 ^min^−1^, which is more than 50 times than that of ZnO nanofibers (4.7 × 10^−4 ^min^−1^), shown in Fig. 3e. Obviously, RhB is started to degraded under visible light with the help of 5 at.% Mn^2+^-doped catalyst (see Fig. 3c), and the rate constant is 4.8 × 10^−3 ^min^−1^, which is 10 times than pure ZnO. Furthermore, the rate constants increase to 1.1 × 10^−2^ and 2.5 × 10^−2 ^min^−1^ after 10 and 15 at.% doping, respectively. To quantize the apparent quantum efficiency (AQE) of all samples, AQE can be defined as follows^31^:


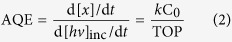


where d[*x*]/d*t* is the initial rate for RhB degradation per unit volume, which equals to *k*C_0_ in our case (C_0_ = 2.5 × 10^−5^ mol/L); d[*hν*]_inc_/d*t* is the total optical power (TOP) radiating on the sample. As the catalyst loading is 1 g/L and the density for ZnO is 5.606 g/cm^3^, the TOP radiating on the nanofibers is 0.01784 mW/mL. Then, the AQE for all samples could be calculated by using formula 2 (see Fig. 3e). In this paper, TOP is defined by the incident photons on the sample surface instead of the photons absorbed by the photocatalyst. Thus, the real quantum efficiency might be even higher than the obtained value. In spite of this there are indeed significant improvement of photocatalytic properties in Mn-ZnO nanofibers. The AQE is 2.47% for the 5 at.% Mn^2+^-doped sample, which improves 10 times compared with pure sample (0.24%). After loading up to 15 at.%, the AQE increases to 12.77%, which is 50 times than that of pure ZnO. This excellent degradation behavior enables ZnO a promising alternative for visible light active photocatalysts.

### Photocatalytic Mechanism

From the bandgap energy point of view, we have studied the improved photocatalytic mechanism of Mn^2+^ doping in the visible light range. Figure 3a represents UV−vis diffuse reflection spectra of ZnO and Mn-ZnO nanofibers. It is noteworthy that there are varying degrees of absorption for all Mn-ZnO samples in the visible light range, which maybe contribute to doping or surface defects. The bandgaps could be obtained from the diffuse reflection spectra using the Tauc plot (inset of Fig. 3a)^36^. Obviously, the ZnO nanofiber sample (3.25 eV) is much higher than Mn^2+^-doped samples, but a little lower than the theoretical value (3.37 eV), which may be affected by the nanometer size effect^19^. And the bandgaps for Mn-ZnO nanofibers are further narrowed by an increased loading (see Table S2). More intuitively, the middle inset of Fig. 3a indicates the color turned to yellow after loading with 15 at.% Mn^2+^ ions. There are probably two reasons for this: One is due to the doping ions in lattice that lower the band gap^17^; another is related to annealing in N_2_ ambient, which introduces N-doping^37^ or large amount of surface defects^7^, such as dangling bonds or oxygen vacancies. It is documented that surface defects are well-known to enhance photocatalytic activity evidently despite various other crystal defects^38^,^39^. Note that surface defects are also associated with a number of states such as zinc interstitials (Zn_i_^++^, Zn_i_^+^, Zn_i_^*^, extended-Zn_i_s (ex-Zn_i_s)), oxygen vacancies (V_O_^++^, V_O_^+^ and V_O_^*^) and zinc vacancies (V_Zn_”, V_Zn_’ and V_Zn_)^40^. To elucidate the photocatalytic mechanism that involves Mn^2+^ doping or rich vacancy defects on the nanofiber surface, we have studied the surface element compositions and chemical states of all samples using XPS analysis and the band gap of Mn^2+^-doped ZnO crystal by first-principles calculations.

**Figure 4:**
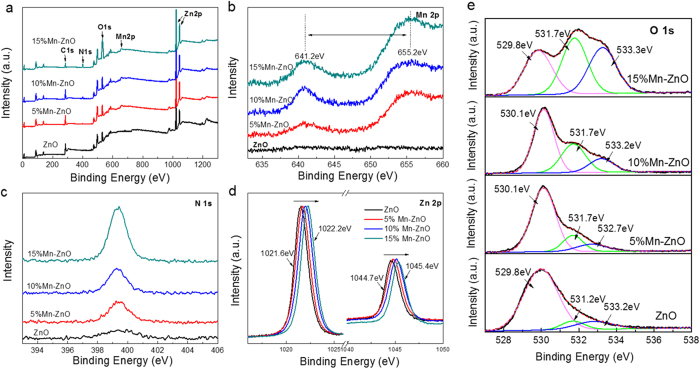
(**a**) XPS spectra of undoped and Mn^2+^-doped ZnO NFs. (**b**–**e**) Mn 2p, N 1s, Zn 2p, and O 1s scan, respectively, of the four samples. The black solid lines are the experimental data, whereas the red dotted lines and thin lines are the fitting results.

For XPS analysis, Zn and O elements are observed in all samples in Fig. 4a, but Mn and N are only detected in Mn-ZnO samples. There is a distinct peak related to N 1s in the 0.15 Mn-ZnO sample, owing to the nitridation on the nanofiber surface. To study the chemical state of each individual element, high-resolution XPS peaks for Mn 2p, N 1s, Zn 2p, and O 1s states are provided. For Mn-ZnO nanofibers (Fig. 4b), a double peak at 641.2 and 655 eV is corresponded to the Mn^2+^-2p level of p_3/2_ and p_1/2_, respectively, indicating that the chemical state of Mn is present as Mn^2+^ in all Mn-ZnO samples. It further confirms that Mn^2+^-doped ZnO nanofibers have been successfully prepared. In Fig. 4c, the core level spectrum of the N 1s region shows a symmetric peak centered at 399.3 eV, which suggests that the N chemical state is present. Because this peak lies in between the typical binding energy for zinc nitride (396–397 eV)^41^ and NO type species (above 400 eV), it can be attributed to the N 1s of oxynitride (O-Zn-N)^18^. This result indicates N atoms occupy at O sites on the ZnO nanofibers surface during annealing, when N_2_ as an N precursor favors the formation of Zn-N bond. Since all samples are calcinated under the same condition, the Mn^2+^ doping seems to facilitate the surface nitridation, which could be attributed to that Mn owns the more chemical states than Zn and thus easier to be nitrogenized^42^,^43^. In addition, doping destabilizes the crystal lattice, leading to an access for N atoms embedded in it. As shown in Fig. 2d, the peaks related to Zn 2p for all samples are clearly observed. There are two symmetric peaks in the Zn 2p region. The peak centered at 1022.2 eV corresponds to Zn 2p_3/2_ and another one centered at 1045.4 is assigned to Zn 2p_1/2_, indicating a normal state of Zn^2+^ in the Mn-ZnO nanofibers. Note that the diffraction peaks of both Zn 2p_3/2_ and 2p_1/2_ shift monotonically with the increase of Mn^2+^ ions, which can be attributed to replacement of Zn^2+^ by Mn^2+^ and an added Zn-O-Mn binding energy^44^,^45^.

Figure 4e displays the high-resolution O 1s spectra of all doped and undoped samples. It shows the asymmetric O 1s core spectrum deconvoluted with three peaks. The first peak centered at 529.9 eV is corresponded to the O^2−^ ions in the wurtzite structure (O-Zn)^46^, while the medium peak at 531.6 eV is assigned to O^2−^ ions in the oxygen deficient regions^47^ and the third peak at 533.2 eV is associated with the presence of chemisorbed oxygen on the surface^48^. Clearly, the O 1s peaks for Mn-ZnO nanofibers become much broaden with a suppression of surface defects^15^. It could be fitted by Gaussian distribution with three peaks which correspond to O-Zn, O-H and O-O, respectively. It is noteworthy that the medium binding energy component, centered at 531.6 eV, is connected to the variations in the concentration of oxygen vacancies, therefore, the intensity changes of this component can be connected in part to the variations in the concentration of surface oxygen vacancies. The intensity of this peak increases with the increasing Mn^2+^ and N concentration, as Table 1 shows. For 0.15 Mn-ZnO nanofibers, the intensity of the peak at 531.5 eV is obviously stronger than that of others, while the area ratio of the peak at 531.6 eV to the one at 529.9 eV is about 1.15, and this ratio is only 0.09 in the ZnO nanofibers. It suggests that the oxygen vacancies in fibrous surface are highly increased after doping with Mn^2+^ while annealing at N_2_ atmosphere; this could partly explains the former bandgap results.

**Figure 5:**
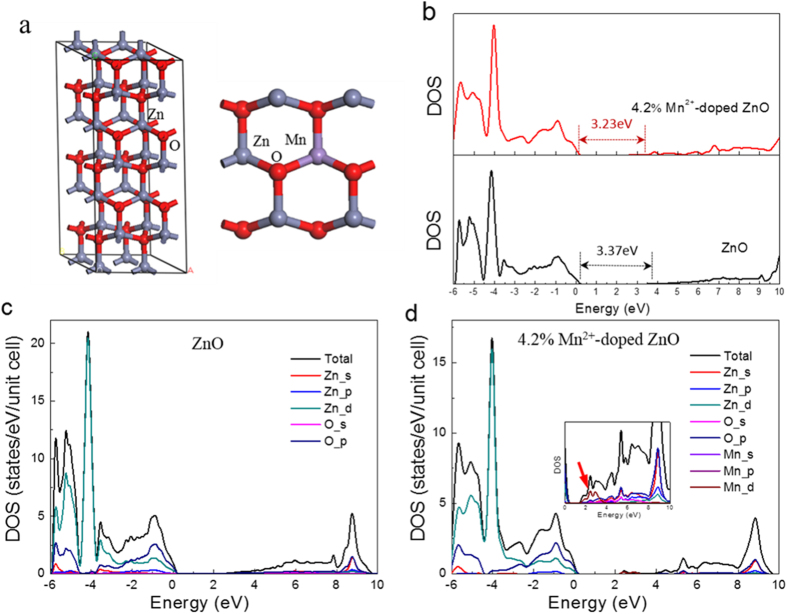
(**a**) 2 × 2 × 3 ZnO supercell with a wurtzite structure and view of the Mn^2+^-doped ZnO (1 0 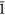 0) surface. (**b**) Calculated DOS of ZnO and 4.2% Mn^2+^-doped ZnO in the form of a bulk crystal. (**c**,**d**) Electron densities of states of bulk ZnO and 4.2% Mn^2+^-doped ZnO. The energy of the valence band maximum of the bulk phase is taken to be zero.

Another possible reason for the gap reduction is the partial replacement of Zn^2+^ by Mn^2+^ in ZnO lattice. To investigate Mn doping effect on orderly ZnO crystal, the bandgap for Mn^2+^-doped ZnO (Mn-ZnO) crystal has been calculated from first-principles using a 48 atom supercell, and the structure of Mn-ZnO (1 0 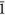 0) surface is shown for replacing one Zn atom with a Mn atom, which corresponds to 4.2 at.% Mn doping, shown in Fig. 5a. Figure 5b shows the total density of states (DOS) for pure and 4.2% Mn^2+^-doped ZnO (0.042 Mn-ZnO), where the Fermi level is set at zero and the “scissor operator” has been set to 1.26 eV. The calculated band gap for bulk ZnO and 0.042 Mn-ZnO crystals after using scissor operator are 3.37 and 3.23 eV, respectively. Thus, it is evident that 4.2% Mn doping could lower the band gap by 0.14 eV, compared with the undoped counterpart. Figures 5c,d depict the partial DOS of states, which indicate that the valence band is mainly contributed by O 2p and Zn 3d states, while the conduction band comes mainly from Zn 4s and O 2p states. In the inset of Fig. 5d, the magnified electron density distributions of the conduction band for 0.042Mn-ZnO sample are shown. Noticeably, neither the Zn 3d and 4s nor the O 2p introduce mid-gap states, the effect state that produces a gap is the Mn 4d (indicated by an arrow). Because the lower-energy mid-gap states are derived from hybridization of the Mn 4d orbital with the Zn 4s and O 2p orbitals, the conduction band tail would produce charge transfer from the Mn 4d orbital to the Zn 4s and O 2p orbitals, which should be responsible for narrowing the band gap. However, this calculated band gap value for bulk 0.042Mn-ZnO is still much higher than the experimental value of 2.5eV for 0.05Mn-ZnO nanofibers (Supplementary Table S2), which displays pale yellow color (Supplementary Figure S3). One of the reason is that ZnO with nanostructure has a narrower band gap nature induced by trap states on the nanofiber surface^4^,^49^, comparing with that of bulk structure. Another reason may be due to the limited effect of Mn doping independently. To observe the individual catalytic effect of Mn^2+^, the Mn^2+^-doped ZnO sample was prepared by annealing under vacuum circumstance. As expected, the photocatalytic properties of this sample is much lower than the Mn^2+^-doped and N-decorated ZnO samples, which confirmed the vital N doping effect for improving photocatalytic activity (see Figure S4).

## Discussion

**Figure 6:**
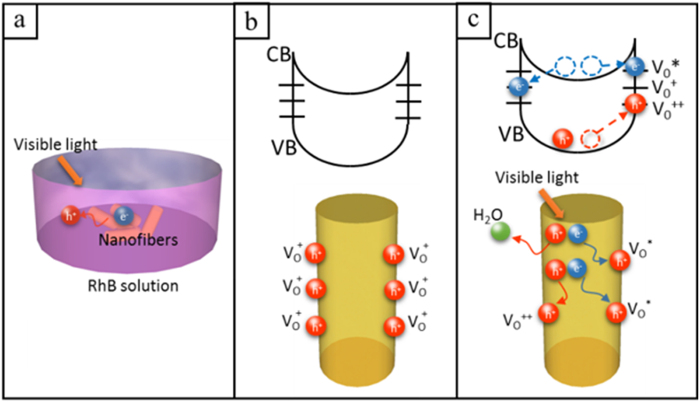
(**a**) Visible light photocatalysis process in NFs. (**b**,**c**) Schematic of trapping and photocatalytic mechanism in a single NF under dark and visible light.

Combined with the above conclusions, the predominant effects on such a significant band gap decreasing for ZnO can be contribute to the high density of surface defects induced by N doping. It is documented that the band gap of a material could be substantially narrowed by introducing defects and disorder. For instance, Chen *et al.*^50^ fabricated the black TiO_2_ nanoparticles with large amounts of defects and disorder introduced by hydrogenating and resulting in a great enhancement of photocatalytic activity. Compared with TiO_2_, it is hard to nitrogenize ZnO under the same conditions. Yang *et al.*^18^ modified it by annealing under ammonia and fabricated N-doped ZnO nanowires. Although N concentration in this sample is only 4%, it shows a significant enhancement in conversion efficiency in the visible region. Since doping introduces more local lattice structure distortion and surface defects, which favors the diffusion of the N atoms, doping with ions make it more easily for N atoms embraced on nanofiber surface. In this work, we demonstrate a 0.15Mn-ZnO nanofiber sample with 10% N concentration in the surface, depending on XPS analysis (Fig. 4c), and show high quantum efficiency in visible-light range. The significant enhanced photocatalytic activities come from the synergetic effects between Mn^2+^ and N, which induce defects states and large amounts of oxygen vacancies. The DFT calculation indicates Mn 4d state that brings in the lower-energy mid-gap states is the effect state for reducing the band gap of ZnO. For N doping effect, it is proved that the oxygen vacancies (V_O_s) in 0.15Mn-ZnO nanofibers surface were highly increased when annealing at N_2_ atmosphere (see the XPS results). Normally, V_O_ exist as V_O_^+^ state and captures charge carriers, thereby delaying the recombination process^13^. Figure 6 shows a schematic of the gain process in photocatalytic property with the presence of rich V_O_ states at the fibrous surface. Here we propose a mechanism to be effective in Mn-ZnO nanofibers that electrons are trapped at the surface oxygen vacancies and multiple holes separate out, then efficiently oxidize the dyes. Upon illumination, when the photocatalysts are excited by photon energy surpassing its band gap, electron-hole pairs are generated and electrons are readily trapped at the V_O_ states, releasing holes from nanofiber surface, which enhance the oxidation reaction with organic pollutants (Fig. 6a). The top schematics in Fig. 6b,c present the energy band diagrams for 0.15Mn-ZnO nanofibers in dark and under illumination. For V_O_ states, it has been shown the following reactions occur in nanomaterials. V_O_^+^ could transform to V_O_^*^ (neutral) and V_O_^++^ states, when the V_O_^+^ captures an electron from CB and forms a neutral state (V_O_^*^), at the same time a V_O_^++^ state is formed by V_O_^+^ captures a hole from VB^51^,^52^, as described by the equations following:


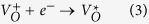



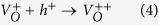


where V_O_^*^ state is 0.86 eV below the CB and V_O_^++^ state is 1.16 eV above the VB, relatively to a typical band gap of 3.37 eV^53^. Figure 6b shows the normal states of V_O_s on fibrous surface under dark. Upon visible-light illumination, not only the V_O_s provide the mid-gap states, which serve as intermediate steps for the photoexcitation process, they also capture electrons from CB, helping the holes release from the catalyst, and exist in the grain boundaries (V_O_^++^), trapping holes at V_O_^++^ and electrons at V_O_^*^, consequently delaying the recombination process (Fig. 6c). Hence, the high level of V_O_^+^, trapping at nanofiber surface, drastically affects the band gap and the quantum efficiency of Mn-ZnO nanofibers. Moreover, Figure S5 illustrates our proposed mechanism of the synergetic effects for Mn^2+^ and N doping in ZnO photocatalyst. The electrons, instead of generating from the VB, could be first excited from N2p levels to the defect energy states, then transfer to the Mn4d states, or excited from N2p levels to Mn4d states directly^54^. Thus, the synergetic interactions can impart multiple charge transfer pathways and long mean free path of charge carriers in ZnO nanofibers, which highly improve the photocatalytic efficiency.

In summary, we have successfully prepared a Mn^2+^-doped ZnO photocatalyst with high density of surface oxygen vacancies via a facile electrospinning method combined annealing under N_2_ ambient. The remarkable enhancement in photocatalytic activity and stable photocatalytic cycles are clearly observed. For 0.15Mn-ZnO nanofibers, the apparent quantum efficiency could reach up to 12.77%, which is 50 times than that of pure ZnO. The significant enhanced visible photocatalytic properties for Mn-ZnO nanofibers are due to the synergetic effects of both Mn^2+^ doping and N decorated. The DFT calculation and XPS spectra indicate that Mn 4d and O-Zn-N bonds induce the huge band gap decrease and thus significantly enhance the absorption of ZnO nanofibers in the range of visible-light. Large density of V_O_s induced by surface N doping provide the mid-gap states, which serve as intermediate steps for the photoexcitation process. Not only they capture electrons from CB, helping the holes release from the catalyst, but also trap holes at V_O_^++^ and electrons at V_O_^*^ at the same time, consequently delaying the recombination process. Further investigations exhibit that the Mn^2+^-doping facilitates the formation of N-decorated and surface defects when annealing in N_2_ atmosphere. Lastly, the method adopted here is not limited to Mn^2+^-doping and can be applied to the other transition metal doping in wide-bandgap semiconductors for visible-light photocatalysis, photoelectrode and photovoltaic cell. Since doping facilitates the nitridation on the surface, we recommend nitriding while doping efficient elements simultaneously such as Fe^3+^, Cu^+^, Ti^3+^, *etc*.

## Methods

### Fabrication of Mn^2+^-doped ZnO nanofibers

Samples of 0, 5, 10 and 15 at.% Mn^2+^-doped ZnO nanofibers were synthesized using an electrospinning approach (identified as ZnO, 0.05Mn-ZnO, 0.1Mn-ZnO, and 0.15Mn-ZnO). Zinc acetate (0.5 g) and manganese acetate were first dissolved in 2 g of deionized water. For chemical reduction of both Mn^4+^ and ZnO, certain amounts of glucose as a carbon source had been introduced to the solution. A PVP (0.4g MW = 1 300 000) solution with a concentration of 8 wt % was then prepared from PVP powders and ethanol with stirring. Afterward, the two prepared solutions were mixed and stirred at room temperature for 2 h. Subsequently, the clear transparent precursor solution was drawn into a hypodermic syringe. The positive voltage of 15 kV and a flow rate of 0.5 mL h^−1^ was applied to the tip and the distance between the needle tip and the collector was 15 cm. Finally, nanofiber samples were removed from the collector plate and taken for thermal treatment in the middle of a furnace tube (volume, 1 L). With a heating rate of 10 °C min^−1^, the prepared samples were annealed under a controlled anoxic environment, which was 50% in air and 50% in nitrogen atmosphere with a flow of nitrogen at 10 ml min^−1^, then after heating to the temperature of 550 °C, the sample would be in pure nitrogen atmosphere. A small amount of oxygen was introduced to decompose PVP, and the second thermal process was carried out for chemical reduction of Mn ions and surface O^2+^.

### Characterization

The external morphologies of the as-prepared nanofibers were examined using a scanning electron microscope (SEM, JSM-7001F, JEOL, Tokyo, Japan), and a transmission electron microscope (TEM, JEM-2010F, JEOL, Tokyo, Japan). The X-ray diffraction (XRD) patterns of all samples were analyzed using a diffractometer (XRD, D/max-2500, Rigaku, Tokyo, Japan) equipped with Cu Kα radiation. Further surface compositions and oxygen vacancies of the Mn-ZnO nanofibers were determined by X-ray photoelectron spectroscopy (XPS, PHI-5300 ESCA, PerkinElmer, Boston, MA).

The photocatalytic activity was evaluated by the degradation of rhodamine B (RhB) dyes under visible light irradiation (λ > 400 nm) using a 300 W Xe lamp with a 400 nm cutoff filter, and the average visible light intensity was 100 mW mL^−1^. The prepared films were immersed in RhB aqueous solution (2.5 × 10−5 M, 10 mL). Before illumination, the RhB aqueous solution was stirred in the dark for 2 h to ensure an established adsorption/desorption equilibrium. During photodegradation testing, the RhB solution with the photocatalyst film was continuously stirred using a dynamoelectric stirrer, and the absorbance of the resulting solution was monitored by colorimetry with a UV−visible (UV−vis) spectrophotometer (Shimadzu, UV3600).

### First-principles calculation

Our calculations based on the first-principles density functional theory (DFT) were carried out by the Vienna Ab initio Simulation Package (VASP). The projector augmented wave (PAW) method was used in the description of exchange-ion interactions. The exchange and correlation functional were described within the generalized gradient approximation (GGA) with the Perdew–Burke–Ernzerhof (PBE). A Monkhorst-Pack k-point mesh of 2 × 2 × 1 was used for irreducible Brillouin zone sampling, while the plane-wave cutoff energy was set to 400 eV. In all calculations, self-consistency was achieved when the total energy converged to less than 1.0 × 10^−4 ^eV/atom and the geometry relaxation tolerance was below 0.01 eV/Å in all forces. On the basis of the wurtzite structure with space group symmetry of P63mc, the 2 × 2 × 3 supercell containing 48 atoms was adopted for pure ZnO. The calculated bulk lattice parameters are a = b = 3.249 Å, c = 5.205 Å, α = β = 90°, and γ = 120°, which are obtained from XRD results. For the Mn-ZnO sample, one Zn atom is replaced by a Mn atom giving the doping molar concentrations of 4.16 at.% (see Fig. 6a).

It is well-known that first-principle calculations tend to underestimate the absolute band gap values of semiconductors^55^. Some authors introduced coulomb interactions between the d electrons or calculated both the spin-polarized band structures and density of states of doped configurations to correct the calculated values^56^,^57^. Since we intended to compare the relative changes in energies induced by Mn^2+^ doping, we applied a simple “scissor operator” to correct the band gap to the experimental value^58^. In the scissor operator method, the conduction band was shifted upwards by 1.26 eV.

## Additional Information

**How to cite this article**: Wang, Y. *et al.* Synergistic effect of N-decorated and Mn^2+^ doped ZnO nanofibers with enhanced photocatalytic activity. *Sci. Rep.* **6**, 32711; doi: 10.1038/srep32711 (2016).

## Supplementary Material

Supplementary Information

## Figures and Tables

**Table 1:** Details of the XPS peak information.

Peak Position (eV)	ZnO		0.05Mn-ZnO		0.1Mn-ZnO		0.15Mn-ZnO	
	Area Ratio	Content Ratio	Area Ratio	Content Ratio	Area Ratio	Content Ratio	Area Ratio	Content Ratio
529.9	1	81%	1	74%	1	56%	1	32%
531.6	0.09	7%	0.21	16%	0.53	30%	1.15	36%
532.8	0.15	12%	0.14	10%	0.25	14%	1	32%
