# Supplementary material for: Synergistic effect of N-decorated and Mn2+ doped ZnO nanofibers with enhanced photocatalytic activity
Source: Sci Rep. 2016 Sep 7;6:32711. doi: 10.1038/srep32711 (PMC5013319; doi:10.1038/srep32711)
Supplement: Supplementary Information [file srep32711-s1.pdf]

Synergistic effect of N-decorated and $Mn^{2+}$ doped ZnO nanofibers with enhanced photocatalytic activity

Yuting Wang[a], Jing Cheng[a], Suye Yu[c], Enric Juan Alcocer[b], Muhammad Shahid [a], Ziyuan Wang[a], Wei Pan[*,a]

[a]State Key Laboratory of New Ceramics and Fine Processing, School of Materials Science and Engineering, Tsinghua University, Beijing 100084, People's Republic of China

[b]Department of materials, Imperial College London, Exhibition Road, London SW7 2AZ, United Kingdom

[c]Department of Physics, University of Science and Technology Beijing, Beijing 100083, China

Corresponding Author

Correspondence and requests for materials should be addressed to W.P. (email: panw@mail.tsinghua.edu.cn)

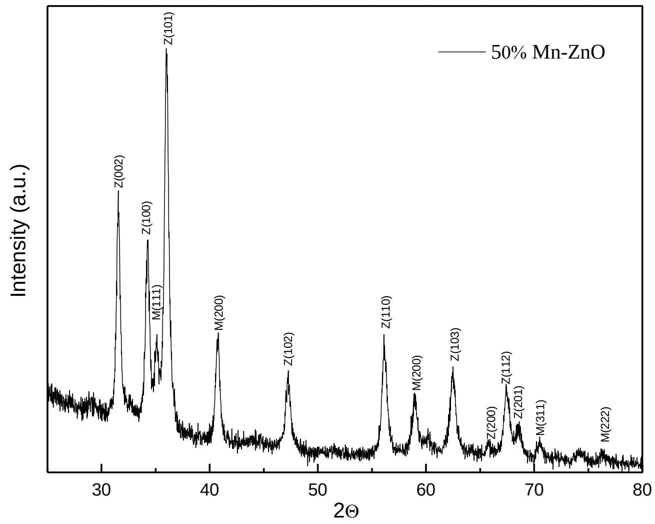


Figure S1. XRD patterns of 50% $Mn^{2+}$-doped ZnO nanofibers.

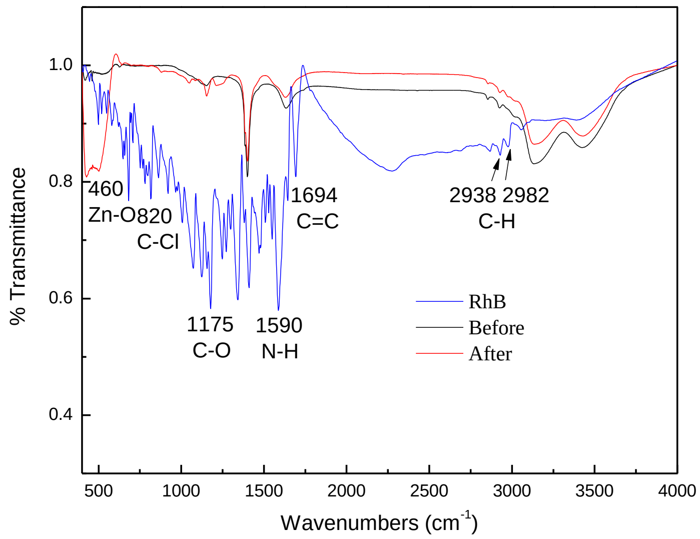


Figure S2. FTIR spectra of RhB, 15%Mn-ZnO NFs before and after photocatalytic cycles.

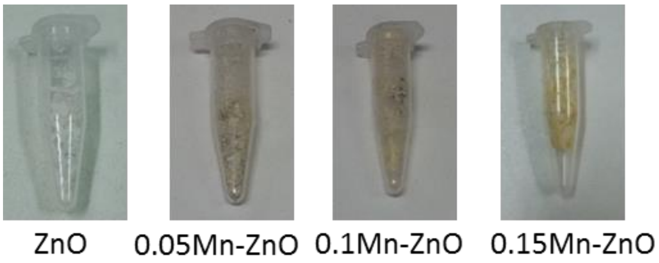


Figure S3. Digital photo images of ZnO, 0.05Mn-ZnO, 0.1Mn-ZnO, 0.15Mn-ZnO NFs powders.

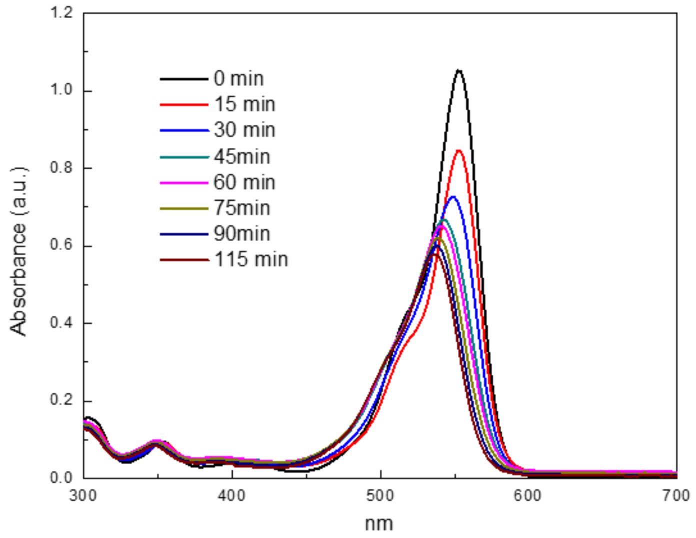


Figure S4. UV-vis absorption spectra of RhB at different time in the presence of 15% $Mn^{2+}$-doped ZnO nanofibers annealed under vacuum.

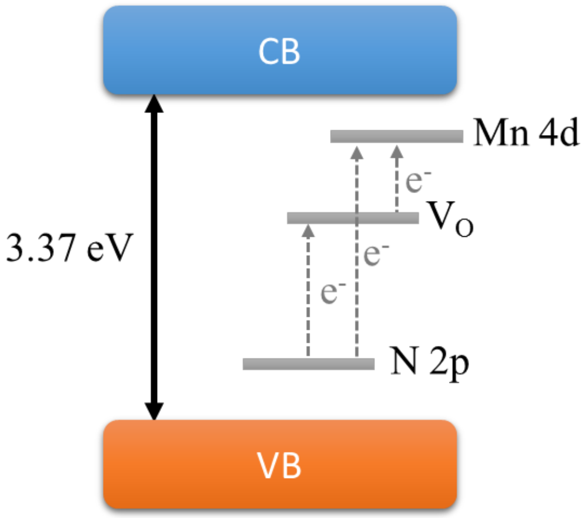


Figure S5. Proposed energy-band structure model for the Mn-doping and N-decorated ZnO photocatalyst.

Table S1 Lattice constants of ZnO and $Mn^{2+}$-doped ZnO nanofibers using XRD.

| | a | c |
|---|---|---|
| ZnO | 3.242 | 5.194 |
| 5%$Mn^{2+}$-doped ZnO | 3.250 | 5.209 |
| 10%$Mn^{2+}$-doped ZnO | 3.251 | 5.211 |
| 15%$Mn^{2+}$-doped ZnO | 3.251 | 5.211 |

Table S2 Band gap values of ZnO and $Mn^{2+}$-doped ZnO samples.

| Sample | Band gap (Experimental) | Band gap (Calculated) |
| --- | --- | --- |
| ZnO | 3.25 eV | 3.37 eV |
| 5%$Mn^{2+}$-doped ZnO | 2.5 eV | 3.23 eV (4.2%) |
| 10%$Mn^{2+}$-doped ZnO | 2.4 eV | - |
| 15%$Mn^{2+}$-doped ZnO | 2.3 eV | - |
